# In silico and in vitro evaluation of the potential genotoxic impurities of vildagliptin

**DOI:** 10.1007/s00210-024-03781-6

**Published:** 2025-01-21

**Authors:** Muhammed Hamitoğlu, Gulcin Tugcu, Ayşe Gökçen Kılıç, Gülşah Esen, Ahmet Aydin

**Affiliations:** Department of Toxicology, Faculty of Pharmacy, Yeditepe University, 34755 Ataşehir, Istanbul Turkey

**Keywords:** Bacterial reverse mutation assay, Clastogenicity, ICH, In silico, Micronucleus test, Mutagenicity

## Abstract

Establishing the safety of impurities in drug substances or products is crucial. The assessment of genotoxicity for these impurities and determining the acceptable limits pose considerable challenges, as recognized in recent guidelines. While the genotoxicity profile of vildagliptin—an oral hypoglycemic drug—is well established, there is limited knowledge about the genotoxic potential of its impurities. In this study, vildagliptin cyclic amidine, vildagliptin diketopiperazine, and vildagliptin amide were assessed in silico and in vitro for mutagenic and clastogenic/aneugenic potential using Ames and micronucleus tests. None of the investigated impurities showed mutagenic or clastogenic potential, thus, are considered non-mutagenic and non-clastogenic/aneugenic in vitro. These findings are consistent with negative in silico predictions for mutagenicity and clastogenicity/aneugenicity in vitro, indicating a good correlation between in silico and in vitro data. In conclusion, this study provides valuable information for the safety assessment of vildagliptin, confirming that its impurities are neither clastogenic/aneugenic nor mutagenic.

## Introduction

Vildagliptin is an oral agent and belongs to a class of hypoglycemic drugs known as dipeptidylpeptidase-4 (DPP-4) inhibitors. Vildagliptin is well tolerated, has a low risk of hypoglycemia, does not cause weight gain, and is administered every day (Chahal and Chowdhury 2007). Vildagliptin has been studied for its effect on islet function as it enhances the glucose responsiveness of both α-cells and β-cells. By inhibiting the DPP-4 enzyme, vildagliptin extends the action of incretin hormones, such as glucagon-like peptide-1 (GLP-1) and glucose-dependent insulinotropic polypeptide (GIP), leading to heightened islet cell sensitivity to glucose levels (Rosenstock et al. 2007).

**Fig. 1:**
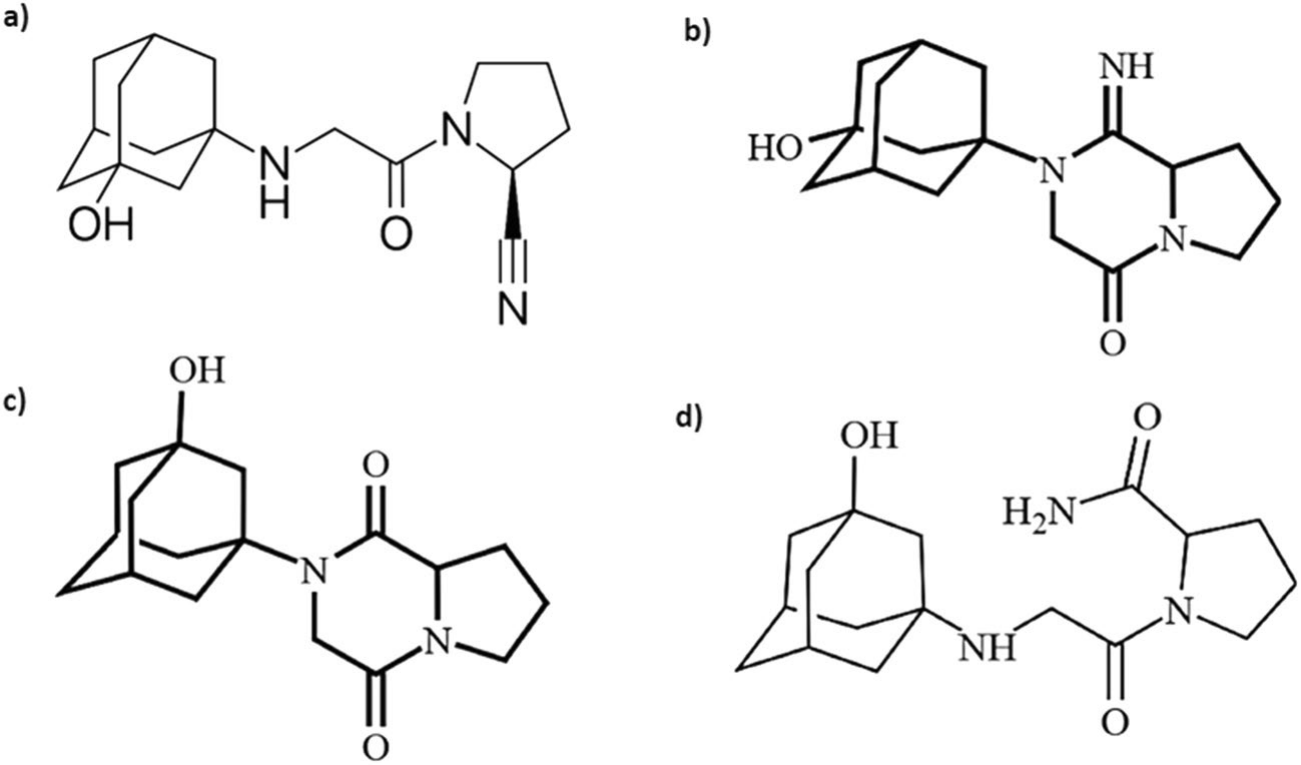
The structure of **a** vildagliptin, **b** vildagliptin cyclic amidine, **c** vildagliptin diketopiperazine, and **d** vildagliptin amide

Structurally, vildagliptin is of the cyanopyrrolidine-4 and α-amino acid amide classes. Vildagliptin stability is influenced by numerous factors, including the presence of additional components in the tablets, whether they are active ingredients or excipients. The vildagliptin molecule includes a secondary amine group, an alcohol hydroxyl in the adamantane fragment, and an amide group bound to the pyrrolidine ring. Examining the structure of vildagliptin reveals vulnerable sites in the molecule, such as the nitrile group, which can undergo hydrolysis to form the amide and then the carboxylic acid (Fig. 1).

The synthesis of vildagliptin begins with the formation of a chiral amine derivative that serves as the amino moiety. This intermediate then undergoes cyclization with a nitrile or similar group to form the essential pyrrolidine ring structure, a step that can introduce impurities due to incomplete ring formation. Subsequent modifications introduce functional groups, most notably the cyanopyrrolidine group, which is critical for Vildagliptin’s DPP-4 inhibitory activity; this step may yield impurities from incomplete functionalization or isomerization. Finally, the product is purified through crystallization or chromatography to ensure the removal of residual by-products or structurally similar impurities (Castaldi et al. 2017).

Impurities in drugs are undesirable substances that have no intended purpose in the drug’s pharmacokinetics or pharmacodynamics. These impurities can originate or are introduced at various stages of the manufacturing process, often stemming from starting materials, reagents, reactive intermediates, side reactions, or degradation (Elumalai et al. 2022). Impurities can result in adverse side effects if they exceed the qualification threshold level. Depending on the daily dose of active ingredient, the qualification thresholds are defined as follows: 1% for < 10 mg daily dose, 0.5% for 10–100 mg daily dose, 0.2% for > 100–2 g daily dose, and 0.15% for > 2 g daily dose. Since the daily dose of vildagliptin is 100 mg, the qualification threshold for its impurities is 0.5%. In this context, it is important to ensure that such impurities are not more toxic than the parent drug.

Generally, impurities should be minimized to meet acceptable safety limits via an effective synthetic process. This process is crucial because the success of a marketed drug depends heavily on achieving maximum therapeutic efficacy with minimal side effects (Teasdale et al. 2013). These concerns highlight the vital importance of conducting rigorous toxicological evaluations, as indicated in the International Council for Harmonisation of Technical Requirements for Pharmaceuticals for Human Use (ICH) guidelines Q3A(R2) and Q3B(R2), which evaluate and ensure the safety of impurities, degradants, and residual solvents in a drug substance or product (ICH Q3A, 2006a; ICH Q3B, 2006b). Two of the most critical toxicological endpoints are mutagenicity and clastogenicity/aneugenicity due to the increased risk of cancer (Franckenstein et al. 2023). The mutagenic and clastogenic/aneugenic potential of a medicinal product is linked not only to the active pharmaceutical ingredient (API), but also to the presence of API degradants in the product or those formed during its production. The assessment and control of DNA reactive (mutagenic) impurities in pharmaceuticals are conducted following the ICH M7(R1) Guideline (ICH 2017). This guideline recommends the use of two complementary models: one statistically based and the other expert rule-based. These methods are essential for evaluating the risk, verifying the elimination, and determining the need to establish control strategies for potentially mutagenic impurities.

The genotoxic safety profile of the parent medicinal molecule vildagliptin has been characterized previously. According to the European Medicines Agency’s (EMA) report, vildagliptin does not indicate a genotoxic potential in several standard genotoxicity tests (EMA 2012). In a cytogenetic study, peripheral lymphocytes were exposed to varying concentrations of vildagliptin for 24 h and 48 h, both timepoints with or without metabolic activation, using chromosomal aberration, sister chromatid exchange, and cytokinesis-block micronucleus assays. The study showed that neither the drug nor its metabolites induced genotoxic effects on the endpoints of the investigated assays (Börçek-Kasurka et al. 2019).

Importantly, even if the parent drug product is not of genotoxic concern, impurities or degradation products might not be non-mutagenic or non-genotoxic. For instance, ranitidine alone is recognized as non-mutagenic, however, previous studies suggest the potential formation of N-Nitrosodimethylamine, which has mutagenic and carcinogenic properties in humans under specific physiological conditions when present in ranitidine-containing products (Franckenstein et al. 2023). Therefore, assessment of the genotoxic potential of both parent compounds and their impurities or degradation products is crucial to ensure the safety of pharmaceutical products.

The degradation of vildagliptin results in the formation of numerous contaminants (Polyakova et al. 2022). Vildagliptin cyclic amidine, vildagliptin diketopiperazine, and vildagliptine amide are identified impurities in vildagliptin preparations as a byproduct or degradation product during the manufacturing or storage process (Fig. 1).

Due to the lack of safety information regarding vildagliptin impurities, specifically vildagliptin cyclic amidine, vildagliptin diketopiperazine, and vildagliptin amide, this study was designed to evaluate their mutagenic and clastogenic/aneugenic potential. The assessment began with a predictive in silico quantitative structure–activity relationship (QSAR) analysis. Additionally, Ames and micronucleus tests (MNT) were conducted in vitro to validate the in silico generated predictions regarding the mutagenic and clastogenic/aneugenic potential.

## Materials and methods

### In silico analysis

The structures of the test compounds were searched for in the PubChem database (https://pubchem.ncbi.nlm.nih.gov/). The compounds’ Simplified Molecular-Input Line-Entry System strings and Chemical Abstracts Service (CAS) numbers were obtained for the in silico evaluation. We considered the prediction of potentially mutagenic impurities using two in silico methods, an expert rule-based method, and a statistics-based method, as recommended by ICH M7 guidelines (ICH 2017).

### Software

We used more than two models to consolidate expert decisions and increase confidence in the predictions. Using additional models can aid in evaluating prediction accuracy and increase confidence in results. Additionally, software, such as QSAR Toolbox, was used to evaluate structural alerts in the molecules. The presence of detailed comments discussing the understanding of the hazard presented by the chemical class can also contribute to the accuracy of the assessment. These comments provide information relevant to the review, such as mitigating features. Therefore, multiple genotoxicity endpoints for vildagliptin and its impurities were evaluated using the following software considered on statistically-based (SB), expert rule-based (RB), or structural alert detection (SA) (Tugcu et al. 2021): US EPA TEST (v.5.1.1) (Toxicity Estimation Software Tool. Washington, DC: United States Environmental Protection Agency) (SB, RB), VEGA-QSAR v.1.2.3 (www.vega-qsar.eu) (SB, RB), ToxRead (v.0.23) (RB, SA), OCHEM Open predictor (Sushko et al. 2011) (SB), ProTox3.0 (Banerjee et al. 2018) (SB), Danish QSARDB (http://qsardb.food.dtu.dk/db/index.html) (SB, RB), and QSAR Toolbox v.4.5 (http://www.qsartoolbox.org/)(SA). Some of these software for toxicity prediction use machine learning algorithms in their QSAR models (e.g., ProTox 3.0), while others are based on read-across methodology (e.g., ToxRead).

### Chemicals and strains

Vildagliptin cyclic amidine (impurity E, CAS No. 1789703–37-2, purity 98%, Batch No. VL/I-927), vildagliptin diketopiperazine (impurity F, CAS No. 1789703–36-1, purity 97%, Batch No. VL/I-931), and vildagliptin amide (CAS No. 565453–39-6, purity 94%, Batch No. VL/I-700) were kindly provided by Helba Pharma company (Istanbul, Türkiye).

*Salmonella typhimurium* bacterial strains and the post-mitochondrial fraction (S9) prepared from rat liver were supplied by the Moltox Molecular Toxicology, Inc. (NC, USA). 2-aminofluorene was obtained from Merck (Hohenbrunn, Germany), and nutrient broth was supplied by Hi Media Laboratories Ltd (Mumbai, India). Histidine was provided by Fluka (USA).

### Mutagenicity assay

The standard plate incorporation Ames test was performed according to the protocol outlined in Organisation for Economic Co-operation and Development (OECD) No. 471 (2020) and Maron and Ames (1983), and in an accredited laboratory by TÜRKAK TS EN ISO/IEC 17025 (No: AB-1764-T). Five histidine-auxotrophic *S. typhimurium* strains (TA98, TA97a, TA100, TA102, and TA1535) were cultured following the respective provider’s instructions. The assay was performed in triplicate, as two independent experiments, in the presence and absence of an external metabolic activation system from Aroclor™ 1254-treated rats (S9 fraction), and at five concentrations (1–5000 µg/plate). Appropriate vehicle controls (methanol: 50 µL/plate) and positive controls (without S9 fraction: 20 µg/plate 4-nitro-o-phenylenediamine for TA98 and TA97a, 1 µg/plate sodium azide for TA100 and TA1535, 0.5 µg/plate mitomycin-C for TA102, and with S9 fraction: 5 µg/plate 2-aminofluorene for TA98, TA97a and TA100, and 10 µg/plate 2-aminoanthracene for TA102 and TA1535) were included in both assays. A positive response was defined as a number of revertant colonies that was at least twice (1.5 for TA102) the number of colonies observed in the negative control.

Notably, for the sake of transparency, it is important to highlight that in the initial Ames test, Vildagliptin amide showed inconclusive results in the TA97a strain with S9 at concentrations of 1 and 10 µg/plate. To ensure reproducibility, follow-up experiments were conducted with all three impurities under the same conditions. These tests confirmed that none of the impurities exhibited mutagenic potential, in line with OECD 471 guidelines. While the follow-up experiments utilized historical positive control data from the initial assay rather than new concurrent controls, the historical values demonstrated sufficient effect size to validate the findings. Additionally, variability in the initial results may have stemmed from conducting assays on separate days, which required averaging control values. To address this, the follow-up experiments were performed on the same day to enhance consistency. The updated data, now included in Table 2, reaffirm the non-mutagenic nature of the investigated impurities.

### Micronucleus assay

The MNT was performed following the OECD guideline (No. 487) for testing chemicals (OECD 2023) in an accredited laboratory (TÜRKAK TS EN ISO/IEC 17025 (No: AB-1764-T)).

Chinese hamster ovary (CHO-K1) cell line (ATCC; CCL-61) was cultured in Ham’s medium F12 supplemented with 10% (v/v) fetal bovine serum (FBS) and 1% (v/v) antibiotics (10,000 U/ml penicillin and 50 mg/ml streptomycin). Cells were cultured at 37°C in a humidified incubator with 5% CO_2_ and were passaged twice a week using 0.25% trypsin solution. Cell culture reagents were obtained from (Gibco, Carlsbad, CA).

Briefly, cells were seeded in 6-well plate at a density of 2 × 10^5^ cells/well for 24 h. After the cells were attached to the surface, they were incubated with different concentrations (100–500 µg/ml) of the compound. After 24 h of exposure, cytochalasin B (3 µg/mL) was added to each well to inhibit cytokinesis for 24 h. Cells were then harvested and fixed twice with methanol acetic acid (3:1) solution. The fixed cell suspension was spread onto pre-cleaned microscope slides and left to air-dry. Cells were stained for 5 min with 5% Giemsa in Sorensen Buffer (v/v). Subsequently, the microscope slides were rinsed in tap water and left to air-dry at room temperature overnight. The potential of the metabolites of vildagliptin impurities to induce micronuclei was also evaluated in the presence of an external metabolic activation system from Aroclor^TM^1254-treated rats (S9-fraction, final concentration of 0.34%) after a 4-h exposure in CHO cells. Appropriate vehicle controls (DMSO: 1%) and positive controls (24 h without S9-fraction: ethyl methanesulfonate, 4 h with S9-fraction: benzo[a]pyrene) were included in all experiments. The number of micronuclei was established by analyzing 1000 binucleated cells per culture per treatment, following the methods previously described by Fenech (2000). Additionally, cells were assessed for the Nuclear Division Index by scoring cells with 1–4 nuclei, using the formula: Nuclear Division Index = M1 + 2(M2) + 3(M3) + 4(M4)/N, where M1–M4 represent the number of cells with 1–4 nuclei, and N is the total number of viable cells analyzed.

The cytotoxicity of the samples was analyzed to establish the concentration levels for MNT. The results showed that all tested samples were not cytotoxic up to 1000 µg/ml. Therefore, the highest concentration of 500 µg/ml was selected for MNT according to the ICH S2 guidelines (2012).

### Statistical analysis

Group comparisons were conducted using SPSS 20 software, with experimental results expressed as mean ± standard deviation (SD). In vitro experiment outcomes were represented as the mean of triplicate measurements ± SD. For comparisons between positive controls and test groups with negative controls in in vitro assays, Dunnett’s multiple comparison test was applied (Dunnett and Crisafio 1995). *p* < 0.05 was considered statistically significant.

## Results and discussion

The safety of pharmaceutical products should be carefully considered, particularly for chronic use where the daily accumulation of impurities could compromise the patient’s health. Official guidelines acknowledge the importance of controlling drug impurities to limit human exposure; hence, knowledge of the toxicity of impurities is essential. To our knowledge, this is the first study to investigate the toxicity of vildagliptin impurities, particularly vildagliptin cyclic amidine, vildagliptin diketopiperazine, and vildagliptin amide. This information is important because diabetes mellitus is a chronic disease associated with tissue damage, specifically diabetic nephropathy, diabetic neuropathy, and diabetic retinopathy. The presence of toxic impurities in drug formulations might compromise or exacerbate the disease.

We assessed the mutagenic and clastogenic/aneugenic potential of the compounds under study using various software packages. Remarkably, all tested compounds were resulted to be negative for all predicted genotoxicity endpoints. Comprehensive information regarding the structures, identity, and toxicity estimation outcomes of both the active substance and its impurities can be found in Table 1.

**Table 1:** Summary of toxicological data (Structural alerts and predictions) for vildagliptin and its impurities

Name	Vildagliptin	Vildagliptin cyclic amidine	Vildagliptin diketopiperazine	Vildagliptin amide
Molecular structure				
SMILES	C1CC(N(C1)C(=O)CNC23CC4CC(C2)CC(C4)(C3)O)C#N	C1CC2C(=N)N(CC(=O)N2C1)C34CC5CC(C3)CC(C5)(C4)O	C1CC2C(=O)N(CC(=O)N2C1)C34CC5CC(C3)CC(C5)(C4)O	C1CC(N(C1)C(=O)CNC23CC4CC(C2)CC(C4)(C3)O)C(=O)N
CAS No	274901-16-5	1789703-37-2	1789703-36-1	565453-39-6
US EPA T.E.S.T. v.5.1.1 Mutagenicity (not mutagenic below 50%)	Negative (0.03)	Negative (0.13)	Negative (0.02)	Negative (-0.10)
VEGA v.1.2.3				
Mutagenicity Consensus of 4 models	Negative	Negative	Negative	Negative
Chromosomal aberration	Positive	Negative	Negative	Negative
In vitro Micronucleus activity Consensus of 2 models	Negative	Negative	Negative	Negative
ToxRead v.0.23 (Ames)	Negative	Negative	Negative	Negative
ToxRead analog compounds				
OCHEM Open predictor (Ames) (probability)	Negative (0.78)	Negative (0.71)	Negative (0.75)	Negative (0.72)
ProTox3.0 (Ames)	Negative (0.69)	Negative (0.68)	Negative (0.68)	Negative (0.69)
Danish QSAR Database				
Ames	Negative	Negative	Negative	Negative
Chromosome aberrations in Chinese hamster lung cells	-	-	-	-
Mutations in Thymidine kinase locus in mouse lymphoma cells	-	-	-	Negative
Mutations in HGPRT locus in Chinese hamster ovary cells	-	-	-	-
Unscheduled DNA synthesis in rat hepatocytes	-	-	-	-
Syrian hamster embryo cell transformation	-	-	Negative	Negative
Sex-linked recessive lethal mutations in rodents	-	-	Negative	-
Micronucleus test in mouse erythrocytes	-	-	-	-
Dominant lethal mutations in rodents	-	-	Negative	-
Sister chromatid exchange in mouse bone marrow cells	-	-	-	Negative
Comet assay in mouse	-	-	-	Negative
Liver specific cancer (rat/mouse in vivo)	-	-	-	-
QSAR Toolbox v.4.5				
DNA binding (OASIS)	No alert	No alert	No alert	No alert
DNA binding (OECD)	SN1 (aliphatic tertiary amine)	SN1 (aliphatic tertiary amine)	SN1 (aliphatic tertiary amine)	SN1 (aliphatic tertiary amine)
Genotoxic carcinogenicity potential (ISS)	No alert	No alert	No alert	No alert
DNA binding alert for Ames, chromosome aberration and MNT potential (OASIS)	No alert	No alert	No alert	No alert
İn vitro Ames mutagenicity alert (ISS)	No alert	No alert	No alert	No alert
in vivo micronucleus mutagenicity alert (ISS)	H-acceptor-path3-H-acceptor	H-acceptor-path3-H-acceptor	H-acceptor-path3-H-acceptor	H-acceptor-path3-H-acceptor
Protein binding alert for chromosome aberration (OASIS)	No alert	No alert	No alert	No alert

The weight of evidence derived from these prediction results strongly suggests that vildagliptin and all assessed impurities are unlikely to manifest bacterial mutagenicity and should not be considered clastogenic/aneugenic in vitro. These findings agree with the experimental results. Notably, Table 1 highlights the detection of an SN1 reaction alert attributed to aliphatic tertiary amine groups, indicating potential DNA binding, rather than a direct mutagenicity hazard alert. This observation extends to both the parent molecule and its impurities. Based on the guideline (ICH 2017), this alert can be neglected and impurities are categorized as Class 4 according to ICH M7 and regarded as not mutagenic impurity. Additionally, another structural alert was flagged across all compounds concerning the in vivo micronucleus test, specifically denoted as H-acceptor-path3-H-acceptor, indicative of the presence of two hydrogen acceptors separated by three bonds. This alert was considered negligible due to its activation by a multitude of substances, while the in vivo MN alerts by ISS profilers identified most chemicals as having structural features linked to positive test results, highlighting the profiler’s poor performance for this specific structural alert (Enoch et al. 2022).

In the Ames assay (Table 2), vildagliptin cyclic amidine, vildagliptin diketopiperazine, and vildagliptin amide were tested at concentrations of 1, 10, 100, 1000, and 5000 µg/plate. No signs of precipitation or cytotoxicity were observed in any strain, with or without metabolic activation. Furthermore, the criteria for a negative mutagenic response were met for all tester strains, both with and without metabolic activation. The mean number of revertant colonies for both negative and positive controls was comparable with historical control ranges for all tester strains, with and without metabolic activation. Furthermore, based on the estimated detection limit for the most relevant mutagens in the Ames test, a minimum concentration of 250 µg/plate will be sufficient to conclude that the impurity poses no concern with respect to genotoxicity (Kenyon et al. 2007).

**Table 2:** Results of mutagenicity assay for vildagliptin cyclic amidine, vildagliptin diketopiperazine, and vildagliptin amide in the *Salmonella typhimurium* strains

		Number of revertant/plate				
		TA98	TA97a	TA100	TA102	TA1535
Without S9						
Negative control		24.0 ± 4.6	146.2 ± 23.8	137.9 ± 5.0	456.5 ± 41.8	14.2 ± 1.8
Positive control		*645.6 ± 48.5	*613.2 ± 73.5	*628.9 ± 75.6	*1110.9 ± 119.6	*625.2 ± 34.1
Vildagliptin cyclic amidine	1	25.3 ± 5.1	137.3 ± 6.7	143.0 ± 17.1	471.0 ± 52.0	13.0 ± 4.0
	10	24.0 ± 7.8	135.3 ± 5.5	134.0 ± 4.6	493.7 ± 46.5	14.3 ± 5.5
	100	23.3 ± 7.6	134.0 ± 8.2	146.3 ± 16.3	487.7 ± 41.1	12.0 ± 2.0
	1000	24.5 ± 3.6	133.0 ± 6.1	141.7 ± 10.4	490.7 ± 44.3	13.7 ± 2.1
	5000	25.3 ± 7.4	135.3 ± 5.5	145.7 ± 30.2	499.3 ± 18.0	11.3 ± 2.5
Vildagliptin diketopiperazine	1	23.7 ± 5.9	145.0 ± 10.5	145.0 ± 15.7	430.7 ± 21.1	13.0 ± 1.0
	10	21.0 ± 1.0	137.7 ± 5.1	132.0 ± 5.3	425.7 ± 23.5	12.7 ± 1.0
	100	21.7 ± 2.3	139.0 ± 8.2	137.3 ± 7.6	438.7 ± 18.8	13.7 ± 3.5
	1000	19.3 ± 3.1	134.3 ± 7.0	139.3 ± 9.0	421.0 ± 17.4	14.7 ± 4.2
	5000	21.0 ± 3.0	144.3 ± 9.9	142.7 ± 3.2	439.7 ± 20.0	14.3 ± 3.1
Vildagliptin amide	1	25.3 ± 2.5	136.3 ± 3.5	131.3 ± 2.1	436.7 ± 10.4	17.3 ± 2.5
	10	25.7 ± 3.2	133.0 ± 7.8	133.3 ± 5.5	430.7 ± 17.2	17.7 ± 2.5
	100	26.7 ± 4.2	135.0 ± 7.2	130.0 ± 2.0	434.3 ± 5.7	17.0 ± 4.0
	1000	28.3 ± 2.3	132.3 ± 5.9	128.3 ± 3.8	444.3 ± 9.3	16.7 ± 2.1
	5000	29.0 ± 1.0	132.3 ± 6.8	130.0 ± 2.0	447.7 ± 23.9	17.7 ± 0.6
With S9						
Negative control		31.8 ± 4.9	196.0 ± 29.2	151.9 ± 1.2	474.5 ± 22.4	12.0 ± 1.6
Positive control		*1591.7 ± 194.1	*653.2 ± 44.8	*1000.0 ± 6.8	*1043.1 ± 41.6	*217.0 ± 37.2
Vildagliptin cyclic amidine	1	26.3 ± 4.0	196.8 ± 19.5	152.7 ± 9.1	460.0 ± 35.8	11.3 ± 1.5
	10	27.0 ± 3.0	198.0 ± 21.7	154.7 ± 8.5	453.0 ± 21.2	10.3 ± 2.5
	100	27.3 ± 1.5	201.5 ± 42.9	150.0 ± 9.2	444.3 ± 30.4	11.0 ± 2.0
	1000	23.7 ± 5.5	188.5 ± 23.4	155.7 ± 5.5	448.0 ± 29.0	10.7 ± 1.2
	5000	27.0 ± 4.6	197.3 ± 28.0	160.0 ± 9.8	461.3 ± 35.5	11.7 ± 3.1
Vildagliptin diketopiperazine	1	29.0 ± 4.4	203.5 ± 11.7	136.3 ± 10.2	498.3 ± 22.5	21.3 ± 2.5
	10	29.7 ± 1.5	200.8 ± 25.0	140.3 ± 3.2	492.0 ± 18.3	19.3 ± 3.1
	100	28.0 ± 3.5	203.3 ± 28.4	141.3 ± 7.4	511.7 ± 34.8	18.0 ± 1.7
	1000	30.3 ± 4.0	209.5 ± 21.7	147.0 ± 10.6	497.0 ± 9.5	18.7 ± 1.5
	5000	28.7 ± 4.0	213.8 ± 19.5	142.3 ± 4.2	509.3 ± 10.6	17.7 ± 3.8
Vildagliptin amide	1	26.7 ± 3.5	196.8 ± 19.5	140.5 ± 3.5	476.5 ± 4.9	20.0 ± 1.4
	10	29.7 ± 2.1	198.0 ± 21.7	142.0 ± 2.8	467.7 ± 18.9	17.5 ± 0.7
	100	28.7 ± 1.5	209.0 ± 33.4	138.0 ± 1.4	471.3 ± 15.0	18.5 ± 2.1
	1000	25.0 ± 3.6	188.5 ± 23.4	143.5 ± 2.1	466.3 ± 5.7	17.0 ± 1.4
	5000	28.7 ± 4.5	197.3 ± 28.0	144.5 ± 2.1	463.7 ± 26.6	19.0 ± 1.4

Dunnett’s multiple comparison test was carried out for statistical analysis. The asterisk (*) indicates *P* < 0.05 as compared to control group. For test groups, *p* > 0.05 versus negative control. For assays conducted without the S9 fraction, positive controls included 20 µg/plate 4-nitro-o phenylenediamine for TA98 and TA97a, 1 µg/plate sodium azide for TA100 and TA1535, and 0.5 µg/plate mitomycin-C for TA102. When the S9 fraction was introduced, positive controls featured 5 µg/plate 2-aminofluorene for TA98, TA97a, and TA100, as well as 10 µg/plate 2-aminoanthracene for TA102 and TA1535. The historical controls range of the testing facility were as follows: TA98(-S9): NC 15–32, PC 476–728; TA98(+ S9): NC 19–34, PC 1370–1810; TA97a(-S9): NC 118–272, PC 513–721; TA97a(+ S9): NC 125–248, PC 565–757; TA100(-S9): NC 128–151, PC 487–728; TA100(+ S9): NC 129–160, PC 862–1050; TA102(-S9): NC 407–548, PC 907–1610; TA102(+ S9): NC 432–537, PC 883–1244; TA1535(-S9): NC 10–27, PC 535–670; TA1535(+ S9): NC 9–24, PC 161–265

Table 3 presents the results of the micronuclei assay in the CHO cell line. Positive controls yielded significant increases in the percentage of cells with micronuclei in both experiments, with or without metabolic activation, indicating a valid assay. The examination of all tested concentrations did not show statistically significant increases in the percentage of micronucleated cells in cultures treated with the test article, and in the concurrent vehicle control under any assay condition.

**Table 3:** Summary results of cytotoxicity and micronucleated cells for vildagliptin cyclic amidine, vildagliptin diketopiperazine, and vildagliptin amide in the micronucleus test

Vildagliptin cyclic amidine	** − S9**	**Samples**	**MN/1000 BN cells (avg ± SD) %**	**NDI (avg ± SD)**
		Negative control	2.03 ± 0.20	1.89 ± 0.10
		500 µg/ml	2.02 ± 0.12	1.84 ± 0.06
		250 µg/ml	2.22 ± 0.08	1.99 ± 0.24
		100 µg/ml	1.69 ± 0.04	1.83 ± 0.15
	** + S9**	Negative control	1.69 ± 0.02	3.58 ± 0.31
		500 µg/ml	1.62 ± 0.06	2.65 ± 0.33
		250 µg/ml	1.80 ± 0.14	3.22 ± 0.29
		100 µg/ml	1.82 ± 0.25	2.86 ± 0.29
Vildagliptin diketopiperazine	** − S9**	**Samples**	**MN/1000 BN cells (avg ± SD) %**	**NDI (avg ± SD)**
		Negative control	3.49 ± 0.73	1.89 ± 0.11
		500 µg/ml	3.19 ± 0.37	1.72 ± 0.12
		250 µg/ml	3.03 ± 0.32	1.80 ± 0.06
		100 µg/ml	4.45 ± 0.30	1.97 ± 0.05
	** + S9**	Negative control	4.19 ± 0.73	2.01 ± 0.10
		500 µg/ml	4.43 ± 0.87	1.74 ± 0.12
		250 µg/ml	3.84 ± 0.89	1.73 ± 0.03
		100 µg/ml	4.28 ± 0.67	1.56 ± 0.06
Vildagliptin amide	** − S9**	**Samples**	**MN/1000 BN cells (avg ± SD) %**	**NDI (avg ± SD)**
		Negative control	3.44 ± 1.05	1.90 ± 0.11
		500 µg/ml	2.66 ± 0.40	2.00 ± 0.16
		250 µg/ml	3.35 ± 1.16	2.12 ± 0.13
		100 µg/ml	1.91 ± 0.38	2.20 ± 0.43
	** + S9**	Negative control	4.20 ± 0.52	1.65 ± 0.19
		500 µg/ml	3.92 ± 0.83	1.91 ± 0.22
		250 µg/ml	2.77 ± 0.27	1.81 ± 0.03
		100 µg/ml	3.71 ± 0.96	1.90 ± 0.33

The MN% in positive control treated cells was 25.4 ± 6.0 and 18.7 ± 3.5 in the experiments with and without S9 activation, respectively. Positive control: ethyl methanesulfonate (450 mM) for the experiment without S9 and benzo[a]pyrene (7.5 µg/ml) for the experiment with S9. Dunnett’s multiple comparison test was carried out for statistical analysis. For test groups, *p* > 0.05 versus negative control. *MN* micronucleated, *BN* binucleated, *NDI* nuclear division index

Taken together, our results show that vildagliptin cyclic amidine, vildagliptin diketopiperazine, and vildagliptin amide exhibited neither mutagenic nor clastogenic/ aneugenic properties in both in silico and in vitro evaluations.

Additional vildagliptin impurities, namely 2-pyrrolidinecarboxamide (impurity V1) and 3-amino-1-adamantanol (impurity V2), were previously investigated for genotoxicity in a single study in the literature. The results obtained from the alkaline comet assay in that study indicated that neither vildagliptin nor its impurities (V1 and V2) caused DNA strand breaks at the tested concentrations (Giordani et al. 2019). However, it should be noted that currently, the comet assay is not considered a regulatory assay for the evaluation of pharmaceutical products.

An EMA report, while not specifying the names of the impurities studied, evaluated vildagliptin impurities requiring toxicological qualification through repeat-dose toxicity and genotoxicity studies. Using a vildagliptin formulation containing 2–3% of vildagliptin impurities, the studies showed no evidence of altered toxicity profiles, indirectly supporting the hypothesis that the vildagliptin impurities are not genotoxic (EMA 2012).

Based on the genotoxicity tests conducted for vildagliptin cyclic amidine, vildagliptin diketopiperazine, and vildagliptin amide, the current study provides a valuable contribution to understanding other impurities with a valid in silico and in vitro data correlation.

For mutagenicity, a valid correlation between predicted in silico data and experimental in vitro data is crucial and has been well established over the last decade with thousands of chemicals and pharmaceuticals tested and published by the European Chemical Agency (ECHA) under the European REACH program. In contrast, there is limited data and correlation of in silico or in vitro data on clastogenicity/aneugenicity for pharmaceutical impurities (Franckenstein et al. 2023). This is partially due to the introduction of the ICH M7 guideline, clastogenicity/aneugenicity testing for impurities is no longer part of the standard battery testing. Therefore, this type of clastogenicity/aneugenicity data for impurities provides essential information and fills a longstanding knowledge and data gap.

Several international guidelines and regional recommendations provide instructions to drug developers and regulatory agencies on the evaluation and control of impurities in drug substances and drug products (ICH Q3A, 2006a; ICH Q3B, 2006b; EMA 2006; FDA 2008). When an impurity reaches a level that necessitates qualification, it becomes the responsibility of the drug developer to establish the safety of the impurity. Ensuring whether an impurity in a drug product poses a genotoxic risk is crucial since there are distinct guidelines for limits in drug products for genotoxic and nongenotoxic impurities. For non-genotoxic impurities, the qualification limit of the drug product is typically 0.15%, while for genotoxic impurities the EMA or FDA guidelines recommend much lower limits in drug substances. The guidance adopts the threshold of toxicological concern approach to establish acceptable limits (ICH M7, 2017). The acceptable risk level for a genotoxic impurity, which is also potentially a carcinogen, is set at 1.5 µg/day—considerably lower than the acceptable levels for impurities that are considered non-genotoxic. Conversely, the impurity level in drug products above these thresholds should be classified as safe with toxicological analysis (Zhu et al. 2014).

In conclusion, based on the results of this study, vildagliptin cyclic amidine, vildagliptin diketopiperazine, and vildagliptin amide are not considered to be clastogenic/aneugenic or mutagenic based on the experimental studies and in silico evaluation.

## Data Availability

All source data for this work (or generated in this study) are available upon reasonable request.
